# Involvement of miRNAs in the Cluster of Metabolic Factors of MetS: Nutrition-Genome-MetS Axis

**DOI:** 10.3390/jcm14124234

**Published:** 2025-06-14

**Authors:** Duygu Ağagündüz, Menşure Nur Çelik, Burcu Deniz Güneş, Büşra Atabilen, Buse Sarikaya, Mehmet Arif Icer, Ferenc Budán

**Affiliations:** 1 Department of Nutrition and Dietetics, Faculty of Health Sciences, Gazi University, 06490 Ankara, Turkey; 2 Department of Nutrition and Dietetics, Faculty of Health Sciences, Ondokuz Mayis University, 55000 Samsun, Turkey; mensurenur.celik@omu.edu.tr; 3 Department of Nutrition and Dietetics, Faculty of Health Sciences, Aydın Adnan Menderes University, 09000 Aydın, Turkey; burcudeniz@adu.edu.tr; 4 Department of Nutrition and Dietetics, Faculty of Health Sciences, Karamanoğlu Mehmetbey University, 70100 Karaman, Turkey; busra.atbln@hotmail.com; 5 Department of Nutrition and Dietetics, Faculty of Health Sciences, Amasya University, 05100 Amasya, Turkey; buse.sarikaya@amasya.edu.tr (B.S.); m.arif.icer@gmail.com (M.A.I.); 6 Institute of Physiology, Medical School, University of Pécs, H-7624 Pécs, Hungary

**Keywords:** microRNA, xeno-miRNA, circulating miRNA, metabolic syndrome

## Abstract

MicroRNAs (miRNAs) are key regulators of gene expression and play essential roles in physiological processes such as cell proliferation, apoptosis, and metabolism. They have emerged as promising biomarkers for the early diagnosis, prognosis, and treatment of Metabolic Syndrome (MetS). In recent years, exosome-derived miRNAs, known as “xeno-miRNAs”, which are derived from food, as well as circulating miRNAs, have emerged as areas of intense research due to their potential effects on metabolic disorders. miRNAs influence fasting blood glucose and insulin resistance through mechanisms such as β-cell differentiation, insulin gene transcription, and PI3K–AKT pathway activation. Additionally, miRNAs play important roles in regulating MetS components, as follows: obesity through adipogenesis and lipogenesis; hypertension through regulation of the renin–angiotensin system and vascular tone; and dyslipidemia by modulating lipid metabolism. Emerging evidence suggests that nutrients such as polyphenols and specific dietary patterns can alter miRNA expression, potentially impacting metabolic health. Understanding the interactions between diet and miRNA regulation offers novel insights into the prevention and treatment of MetS. This review explores the mechanisms by which miRNAs influence MetS components, and highlights the growing potential of nutrient-regulated miRNAs as therapeutic targets within the framework of precision nutrition and personalized metabolic disease management.

## 1. Introduction

Metabolic Syndrome (MetS) encompasses a cluster of interrelated metabolic disorders, including central obesity, hypertension, dyslipidemia, impaired glucose regulation, and type 2 diabetes mellitus (T2DM) [1]. The development of MetS is shaped by a combination of genetic predisposition, environmental influences, and lifestyle choices. Among these, diet significantly affects metabolic health by regulating gene expression through epigenetic modifications [2,3]. One notable group of epigenetic regulators, microRNAs (miRNAs), has attracted growing interest due to their regulatory influence on metabolic pathways linked to MetS [4]. These small, non-coding RNAs are increasingly recognized as valuable non-invasive biomarkers, with potential clinical applications in both the early detection and treatment of MetS [5].

miRNAs govern essential cellular functions such as proliferation, apoptosis, and cell differentiation by modulating gene expression at the post-transcriptional stage [6]. In MetS, distinct miRNA expression patterns significantly influence critical biochemical pathways, especially those related to insulin resistance, inflammation, blood pressure regulation, and lipid processing [7]. MiRNAs are suggested to modulate MetS through interconnected pathways that influence key metabolic processes, with the following proposed as major mechanisms in its pathophysiology [8]:

i. Glucose homeostasis regulation—miRNAs influence neural signaling between the brain, muscle, liver, pancreas, and gastrointestinal tract. miR-132 regulates cAMP Response Element-Binding Protein (CREB) in the brain, involved in glucose homeostasis [9]. miR-9 and miR-124a also impact pancreatic beta-cell function, affecting insulin secretion by targeting transcription factors such as One Cut Homeobox 2 and Forkhead Box A2 [10]. miRNAs regulate insulin production, secretion, and sensitivity [11,12]. miR-375 influences beta-cell function and insulin exocytosis, while miR-124a and miR let-7b affect glucose regulation by repressing myotrophin [11]. Elevated glucose levels upregulate miR-30d, thereby enhancing insulin gene expression [12];
ii. Adipogenesis and lipid metabolism—miRNAs regulate adipogenesis and lipid metabolism. miR-143 promotes adipocyte differentiation [13,14], while miR-103 enhances key markers like Glucose Transporter Type 4 (Glut4), Fatty Acid-Binding Protein 4 (Fabp4), and Peroxisome Proliferator-Activated Receptor Gamma 2 (Pparγ2) [15]. The miR-17-92 cluster promotes adipocyte differentiation by inhibiting Retinoblastoma 2/p130 [16]. In obesity, chronic inflammation alters miRNA expression, leading to a loss of miRNAs maintaining metabolically active adipocytes, contributing to obesity and metabolic disorders [8,17]. miR-30a-5p and miR-195 regulate hypothalamic brain-derived neurotrophic factor secretion, which influences appetite and energy balance.
iii. Blood pressure mechanism—miRNAs play a central role in the development and regulation of hypertension. MiR-122 regulates hypertension by modulating the renin–angiotensin system (RAS), endothelial dysfunction, vascular fibrosis, autophagy, pulmonary vascular remodeling, and fibrogenesis through pathways like Nuclear Factor Kappa B (NF-κB), Transforming Growth Factor Beta (TGF-β), β-catenin, Phosphoinositide 3-Kinase/Protein Kinase B (PI3K-Akt), and cationic amino acid transporter [18,19,20,21,22]. MiR-204 inhibits the Src family kinases (SRC)/Signal Transducer and Activator of Transcription 3 (Stat3) module, regulating pulmonary hypertension [19]. The miR-143/145 cluster is a major player in the pathophysiology of hypoxia-induced pulmonary hypertension, which is prevented by miR-143 [20]. MiR-181 targets genes regulating blood pressure, contributing to hypertension, and miR-663 reduces blood pressure by controlling renin gene expression [21]. MiR-155 modulates the TGFβ signaling pathway, influencing cardiac hypertrophy and systemic hypertension [22].

Circulating miRNAs and dietary exosomal miRNAs (xeno-miRNAs) are increasingly acknowledged for their significant influence on metabolic pathways, as they mediate intercellular communication and help maintain metabolic balance [23,24]. Increasing evidence highlights the intricate interaction between circulating miRNAs, xeno-miRNAs, and MetS components, revealing their critical role in disease pathophysiology [5,7,23,25,26,27].

Circulating miRNAs, which are naturally produced and present in biological fluids like blood, saliva, and urine, are considered promising biomarkers for MetS owing to their tissue-specific expression patterns and correlation with important clinical and pathological indicators [27,28]. They regulate essential metabolic processes, including glucose metabolism, lipid homeostasis, and adipogenesis, making them valuable indicators for diagnosing and monitoring MetS progression [25]. The dysregulation of various microRNAs—including miR-33a/b, miR-21, miR-34a, miR-378, miR-107, miR-103, miR-146a, and miR-122, which are involved in regulating glucose metabolism, inflammatory signaling, and adipocyte differentiation—has been associated with the onset and progression of MetS [5,29,30]. Dysregulation of these miRNAs exacerbates hallmark features of MetS, including dyslipidaemia, insulin resistance, and impaired glucose metabolism, ultimately increasing the risk of T2DM and cardiovascular disease [27,31,32].

While endogenous miRNAs play essential roles in various physiological processes, recent studies have highlighted the existence of exogenous dietary miRNAs, known as xeno-miRNAs [33]. Xeno-miRNAs enter circulation via intestinal epithelial absorption mechanisms such as receptor-mediated endocytosis, transmembrane transporters, and phagocytosis [34,35]. Once absorbed, these miRNAs can modulate endogenous gene expression by targeting metabolic pathways related to insulin sensitivity, lipid metabolism, and inflammation [36]. Plant-derived miRNAs, including miR-156, miR-168a, and miR-172a, have been demonstrated to affect protein expression and metabolic processes, potentially contributing to metabolic health [37,38], while animal-derived miRNAs including elevated levels of miR-99a, miR-1, miR-206, and miR-133a-3p have been linked to the formation of lipid droplets in the livers of mice, along with disruptions in glucose and insulin metabolism, underscoring their important role in the progression of metabolic diseases [39].

The growing understanding of miRNA-mediated regulation highlights their potential as both biomarkers and therapeutic targets in MetS management [5,33]. Consequently, exosomal miRNAs emerge as promising candidates for diagnostic and therapeutic strategies aimed at mitigating metabolic disorders [24,40,41]. This study investigates the involvement of food-derived xeno-miRNAs and circulating miRNAs in the cluster of metabolic factors associated with MetS. We aim to investigate how dietary and endogenous miRNAs influence MetS pathophysiology, highlighting their biomarker potential and regulatory effects on metabolic processes.

## 2. Food Xeno-miRNAs and Circulating miRNAs in MetS

miRNAs are crucial regulators of gene expression, influencing metabolic disorders such as MetS through their role in key biological processes [42]. Recent research highlights the importance of both food-derived xeno-miRNAs and circulating miRNAs in modulating metabolic pathways, offering potential for novel diagnostic and therapeutic strategies [7,23,26].

### 2.1. Biogenesis of miRNAs

The biogenesis of miRNAs involves sequential nuclear and cytoplasmic processing [42]. In the nucleus, RNA polymerase II transcribes primary miRNAs (pri-miRNAs), which are subsequently processed into precursor miRNAs (pre-miRNAs) by the Drosha-DGCR8 complex [43,44,45,46]. Exportin 5 transports the pre-miRNAs to the cytoplasm, where the Dicer enzyme processes them into mature miRNAs. These mature miRNAs subsequently bind to the RNA-induced silencing complex (RISC), enabling the regulation of gene expression by promoting mRNA degradation or blocking translation [43,44]. While miRNAs primarily function in post-transcriptional gene regulation, they are also found in multiple cellular compartments, including the nucleus, mitochondria, and nucleolus [47].

Circulating miRNAs are secreted into biological fluids through exosomes, microvesicles, apoptotic bodies, RNA-binding proteins, and lipoproteins, facilitating intercellular communication and systemic metabolic regulation [48,49,50]. Due to their stability and resistance to enzymatic breakdown, these molecules represent promising biomarkers for metabolic disorders [7].

### 2.2. Food-Derived Xeno-miRNAs

Studies suggest that dietary miRNA levels in humans fluctuate in response to food intake [51,52,53,54]. Bioinformatic analyses suggest that animal-derived dietary miRNAs could participate in cancer-related pathways, whereas plant miRNAs demonstrate regulatory effects on metabolism [51]. Certain foods, such as poultry (miR-206), pork (miR-1), rapeseed (miR-156), wheat (miR-156a), barley (miR-168-5p), and corn (miR-319b), are particularly rich in miRNAs, whereas milk and cheese contain lower miRNA levels [55,56]. Moreover, specific miRNAs are found in other foods, such as gga-miR-92-3p, gga-miR-2188, and gga-miR-30c-5p in eggs [57], bta-miR-154c in beef [58], and miR-92, miR-125a, miR-223, miR-let-7c, miR-181, miR-21, miR-155-5p/miR-29b-5p, miR-17, and miR-148a-3p in milk [59,60,61,62,63]. Human milk contains miR-27b, miR-125b, miR-181a and b, and miR-155 [64,65]. Green and yellow vegetables, such as peas, green beans, broccoli, corn, and carrots, are rich in miR-156a [66], while bra-miR156g-5p (56), miR-168, and hvu-miR-168-3p are found in rice [67,68]. Ginger contains miR-168, and miR-167a, miR-1078, miR-34a, miR-143, and miR-145 [69,70,71]. Apple contains mdm-miR-7121d-h [72] and soybean contains miR-159a [73].

### 2.3. Stability and Absorption of Food Derived Xeno-miRNAs

The stability and absorption of dietary miRNAs remain areas of ongoing investigation, with numerous studies highlighting their unique biochemical properties and potential for gene regulation in host organisms [74]. Although dietary miRNAs have been detected in biological samples, research suggests that miRNAs can withstand processing and digestion [75].

A key factor supporting miRNA stability is their encapsulation within extracellular vesicles, such as exosomes, which protect them from enzymatic degradation and facilitate their transfer across biological barriers like the blood–brain barrier and placenta [76,77,78]. Breast milk extracellular vesicles, for instance, have been demonstrated to deliver miRNAs that play a role in immune development, suggesting that dietary miRNAs could exert functional effects when effectively delivered [79,80].

Recent evidence suggests that plant-derived miRNAs can resist degradation during gastrointestinal digestion. Their stability is enhanced by the food matrix and structural modifications such as 2′-O-methylation [81]. For instance, miRNAs in honey such as miR-30c-5p have demonstrated significant resilience under in vitro digestion conditions [82]. Furthermore, dietary miRNAs are taken up by intestinal epithelial cells via caveolin- and clathrin-mediated endocytosis, facilitated by specific membrane receptors [81]. However, circulating miRNAs are reported to remain stable under various storage conditions and are not significantly influenced by food intake [83]. These observations support the hypothesis that dietary miRNAs follow a metabolic absorption curve, although their physiological role post-absorption remains to be fully elucidated [84].

Some bioactive dietary compounds, such as resveratrol, have been found to influence miRNA expression, reducing miR-196b/miR-1290 in leukemia cells, suggesting potential antitumor properties [85,86]. However, the biological function of these absorbed miRNAs in mammalian systems requires further validation. Notably, distinctions have been proposed between endogenous miRNAs and absorbed dietary miRNAs, emphasizing the need to understand exosome secretion, transport, and target interactions [87].

### 2.4. Metabolic Effects of Food-Derived Xeno-miRNAs

The food-derived xeno-miRNAs have been considered to influence metabolic pathways, thereby playing a role in the modulation of various physiological processes. For instance, rice-derived miR-168 has been reported to decrease plasma low-density lipoprotein (LDL) clearance, potentially contributing to dyslipidaemia and increased cardiovascular risk [88,89]. Similarly, hvu-miR-168-3p, derived from rice aleurone, enhances glucose uptake by upregulating glucose transporter 1 (GLUT1), thereby lowering blood glucose levels [68]. Additionally, miR-22-3p inhibits the Wnt signaling pathway, preventing glucose intolerance and T2DM onset, while milk-derived miRNAs, like miR-148a and miR-30b, play a role in controlling lipid metabolism and obesity [90,91].

Rats fed freely for three months exhibited notable changes in miRNA levels depending on their diet. Specifically, miR-27, miR-122, and miR-451 were significantly reduced, while miR-200b, miR-429, and miR-200a were elevated in animals fed with a high-fat diet, a standard diet supplemented with high fructose, or a combination of high-fat and high-fructose diets. Furthermore, a marked decrease in miR-21 expression was observed exclusively in the groups consuming fructose-enriched diets [52].

The effects of plant- and animal-derived xeno-miRNAs on human gene expression and metabolism have been widely reported in the literature. For example, osa-miR168a, originating from rice, targets the human LDLRAP1 gene and has been shown to reduce LDL clearance in the liver, potentially influencing lipid metabolism [88]. Similarly, ath-miR156a, found in vegetables like spinach, lettuce, and cabbage, targets JAM-A in humans, and has been reported to reduce monocyte adhesion triggered by inflammatory stimuli [66]. From animal sources, bta-miR-148a, a bovine-derived miRNA, targets DNMT1 in humans, affecting epigenetic regulation and maintaining Th17/Treg cell balance [92]. Another notable example is miR2911 from honeysuckle, which possesses 28 binding sites in the SARS-CoV-2 genome and has been implicated in inhibiting viral replication [93]. Multiple plant miRNAs, including miR168 and miR156 from strawberry, bol-miR874 from cabbage, and osa-miR168 from rice, have been found to target the TLR3 gene in humans, thereby suppressing inflammatory responses [94]. Furthermore, gma-miR159 from soybean inhibits TCF7, which may suppress breast cancer cell proliferation [95], while gma-miR4995, also from soybean, targets MALAT1 and NEAT1, two long non-coding RNAs involved in tumor progression, thereby exerting anti-tumor effects [96].

### 2.5. Circulating miRNAs and Their Role in MetS

Circulating miRNAs exhibit cell- and tissue-specific expression profiles and play a crucial role in metabolic homeostasis [97]. These miRNAs mediate important events in the pathogenesis of MetS. They are actively released by viable cells or released into the bloodstream as a result of cell death [40].

Alterations in circulating miRNA profiles have been associated with factors that increase the risk of MetS, including insulin resistance, chronic inflammation, and dyslipidaemia [5,31,98]. For example, miR-122 plays a crucial role in hepatic lipid metabolism by regulating lipid synthesis and uptake [31]. miR-107 and miR-103 play a role in glucose homeostasis and insulin signaling [31], while miR-146a modulates NF-κB signaling, influencing inflammatory mechanisms and immune cell activation [99]. miR-146a also modulates glucose-stimulated insulin secretion and cell differentiation [99]. Various miRNAs, like miR-378/378, miR-27a/b, miR-34a, and miR-21, contribute to various aspects of lipid metabolism. Fatty liver disease and dyslipidemia have been linked to the dysregulation of these miRNAs [100].

**Figure 1:**
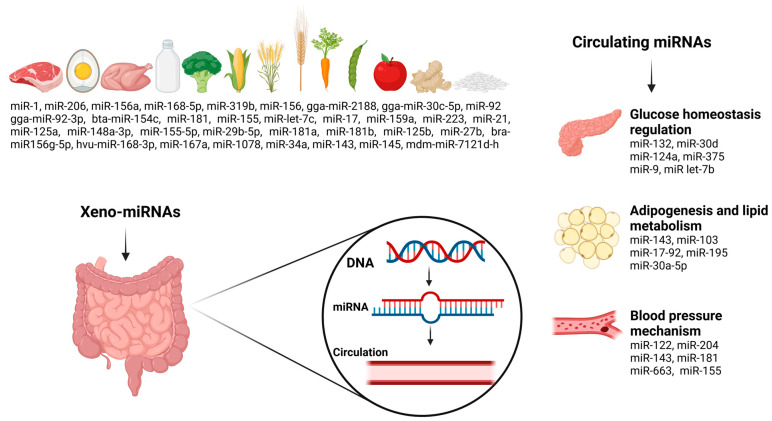
Food xeno-miRNAs and circulating miRNAs in relation to metabolic factors in MetS.

Furthermore, miR-375, miR-16, miR-27a, miR-146a, miR-150, miR-320a, miR-155, miR-15a, and miR-30a-5p have been implicated in T2DM pathogenesis [5,101,102,103,104]. Various miRNAs, such as miR-7, miR-375, and miR-124a, are involved in the development of β-cell and insulin secretion [105,106]. miRNAs have also been shown to affect insulin resistance. For example, miR-320, miR-29, and miR-384-5p modulate components of the insulin signaling pathway, such as PI3K, Insulin Receptor Substrate (IRS) proteins, and the Insulin-like Growth Factor 1 Receptor (IGF-1R) [106]. A comprehensive analysis of miRNA expression in human β-cells and pancreatic islets revealed 384 unique miRNAs, of which 40 were predominantly expressed in islets [107]. Similarly, miR-199a-3p, miR-34a, miR-652-3p, miR-139, miR-223-3p, miR-30d, miR-16-5p, miR-18a-5p, miR-122, and miR-106a-5p have been linked to cardiovascular complications associated with MetS [108,109,110,111]. Elevated levels of miR-34a have been observed in patients with coronary artery disease, exhibiting a negative correlation with silent information regulator 1 (SIRT1) protein expression, thereby promoting endothelial senescence [108]. Numerous miRNAs have demonstrated inverse correlations with biomarkers associated with adverse clinical outcomes. In patients exhibiting the progression of heart failure within 48 h of hospitalization, several biomarkers were identified in association with specific miRNAs, miR-652-3p (soluble ST-2), miR-199a-3p (procalcitonin and galectin-3), miR-223-3p (growth differentiation factor 15), miR-18a-5p (procalcitonin), miR-106a-5p (creatinine), and miR-16-5p (C-reactive protein), implying their potential roles in the molecular pathways underlying cardiac dysfunction [109]. An elevated miR-122-5p/133b ratio has been significantly associated with poorer event-free survival in patients with ST-elevation myocardial infarction (STEMI), corresponding to an approximately ninefold increased risk of mortality or recurrent myocardial infarction and a fourfold greater likelihood of adverse cardiovascular outcomes, making it a potential prognostic biomarker for identifying high-risk patients after primary percutaneous coronary intervention [110]. Inhibiting miR-139-5p restores endothelial function by enhancing angiogenesis and blood perfusion. Its involvement in diabetic vascular complications is attributed to the inhibition of the c-Jun–vascular endothelial growth factor/platelet-derived growth factor-B signaling axis, leading to reduced endothelial cell viability and highlighting its potential as a therapeutic target [111]. The relationship between food-derived xeno-miRNAs, circulating miRNAs, and MetS factors is illustrated in Figure 1.

Clinical studies further support the association between circulating miRNAs and metabolic diseases, with myocardial infarction, heart failure, and atherosclerosis being linked to altered circulating miRNA levels [112,113]. Research indicates that cardiac injuries, including myocardial infarction, result in increased circulating levels of heart-specific miRNAs, such as miR-499, miR-208, miR-1, and miR-133 [113,114]. In contrast, diseases linked to MetS, including coronary artery disease and T2DM, frequently exhibit reduced levels of endothelial-derived miRNAs like miR-126 [113]. Table 1 summarizes key miRNAs affecting components of MetS.

## 3. miRNA in the MetS Hallmarks

### 3.1. Impaired Fasting Glucose

A major factor contributing to impaired fasting glucose in patients with MetS is insulin resistance. A breakdown in insulin signaling under metabolic stress can lead to persistent systemic hyperinsulinemia and insulin resistance, which is characterized as a reduction in the impact of insulin on important target tissues (including adipose tissue, liver, and skeletal muscle). The fundamental reasons behind insulin resistance linked to obesity are complex [115]. Obesity contributes to insulin resistance through multiple interrelated mechanisms. Increased free fatty acid levels can alter insulin receptor substrate-1 (IRS-1), which can disrupt insulin signaling. Adipokine imbalances—such as decreased adiponectin and increased leptin—further reduce insulin sensitivity. Ectopic fat accumulation in the liver, muscle, and heart disrupts glucose metabolism, while chronic inflammation driven by immune cell infiltration in visceral adipose tissue exacerbates insulin resistance. Endoplasmic reticulum stress, mitochondrial dysfunction, oxidative stress, and reduced hepatic insulin clearance are further contributing factors. Moreover, alterations in gut microbiota may lead to increased systemic inflammation via bacterial lipopolysaccharide translocation. Gene expression associated with metabolic dysfunction may also be regulated by epigenetic modifications, such as DNA methylation and histone modifications [115,116,117].

Recent genomics research has demonstrated that miRNAs control gene expression and have a role in the development of insulin resistance and diabetes in MetS [106,118]. miRNAs participate in controlling the synthesis, secretion, and signaling pathways of insulin-related proteins. There is evidence that miRNAs may be important regulators and biomarkers in the early phases of the development and management of insulin resistance [119]. It has been discovered that prediabetic or type 2 diabetic patients have decreased levels of circulating miR-126, miR-15a, miR-223 and miR-29b, which may be useful in predicting the onset of diabetes [120]. Another study discovered that miRNAs (miR-let-7c, miR-30a, miR-122, miR-139 and miR-126) were shown to have elevated plasma levels in patients with MetS and insulin resistance and/or elevated fasting plasma glucose [25]. Also, it was stated that the miRNAs potentially linked to insulin resistance in MetS include miR-375, miR-320b, miR-148a-3p, miR-197-3p, miR-122-5p, miR-19b-3p, miR-342-3p, miR-103, miR-155-5p, and miR-130a [121], and insulin resistance can be managed by using these miRNAs as biomarkers. The majority of miRNAs target several genes, which supports both their usage as biomarkers and their potential use as therapeutics [122]. Findings indicate that adding miRNAs to the ROC curve in addition to traditional indicators like HbA1c and fasting blood glucose can improve diagnosis, help track treatment progress, and be used to evaluate treatment efficacy [123]. In a related study that established a multi-parameter diagnostic model for the early identification of type 2 diabetes using miR-130a, miR-148b, miR-19a and miR-223, the validation set’s AUC was 0.88, and the sensitivity and specificity were 78.36% and 87.63%, respectively [124].

miRNAs may contribute to insulin resistance in MetS by influencing insulin production, mature β-cell function, and β-cell differentiation. The overexpression of islet-specific miR-375 inhibits the insulin gene transcription and glucose-stimulated insulin production [117]. miR-375 modifies secretion via a mechanism that is directly related to insulin exocytosis and unrelated to changes in intracellular Ca^2+^ signaling or glucose metabolism. It was confirmed that miR-375 targets myotrophin (Mtpn) [125]. Due to its potential role in β-cell function regulation, miR-30d may be associated with insulin resistance. A study investigating the circulating levels of specific miRNAs in Indian type 2 diabetic patients found that miR-30d was significantly higher in patients compared to healthy controls [126]. In β cells, glucose-induced insulin production has been shown to be negatively modulated by another miRNA, miR-7. It has been demonstrated that miR-7a2 affects insulin granule fusion with the plasma membrane by targeting the genes involved in insulin synthesis and release [122]. The Mir-200 miRNA family—miR-200c, miR-200a and miR-200b—and miR-141 and miR-429 have been linked to both β-cell dedifferentiation and protection against β-cell apoptosis. The ablation of all miRNAs in this family led to increased protection against β-cell apoptosis, but the deletion of miR-200c, one of the most abundant miRNAs, provides some protection against β-cell apoptosis following oxidative stress [127]. A considerable number of genes are controlled, either directly or indirectly, by miR-34a, which also suppresses the production of the anti-apoptotic protein Bcl-II, resulting in decreased insulin secretion and cell apoptosis [128]. miR-29a is highly expressed in the β cells that secrete insulin. Although miR-29a is necessary for regular exocytosis in pancreatic β cells, elevated levels are linked to impaired β-cell activity [129]. In addition, miR-184, miR-124a2, miR-185-5p, miR-24 and miR-204 have also been connected to the management of insulin production and β-cell proliferation during insulin resistance [117]. Insulin secretion is remarkably influenced by miR-184. It has been found that this miRNA directly targets the mitochondrial glutamate carrier Slc25a22. Slc25a22 suppression reduces the insulin level secreted by pancreatic β cells [122]. The upregulation of miR-204 suppresses the synthesis of insulin by adversely affecting MafA, a transcription factor necessary for insulin transcription [127].

The presence of chronic low-grade inflammation and insulin resistance in MetS is influenced by a variety of microRNAs that facilitate the polarization of macrophages from an anti-inflammatory M2 phenotype to a proinflammatory M1 phenotype [130]. miR-30 suppression increases pro-inflammatory cytokine production and M1 macrophage polarization. The upregulation of miR-1249-3p also reduced pro-inflammatory cytokine molecule expressions, such as IL-6, IL-1β and TNF-α [117]. Insulin resistance and systemic inflammatory incidence are positively correlated with elevated levels of miR-34a and miR-122 and reduced levels of miR-26a and miR-126-3p expression [131,132]. A decreased expression of miR-1934 raises the levels of TNF-α, IL-1β and IL-6. Similarly, the reduced expression of miR-146-a stimulates the inflammatory genes’ expressions after an increase in NF-kB [131]. In reaction to obesity, adipose tissue macrophages release more miR-155, which targets PPARγ and inhibits insulin signaling in muscle cells, hepatocytes, and adipocytes. These findings point to a mechanism by which insulin resistance and inflammation development are impacted by obesity-mediated elevations in miR-155 [133]. Similarly, by blocking insulin signaling, extracellular miR-27a elevation in response to obesity causes insulin resistance. Obesity-induced extracellular miR-27a inhibits the expression of the transcription factor peroxisome proliferator-activated receptor γ (PPARγ), which inhibits the production of inflammatory cytokines [133,134].

Following its synthesis and release from the pancreas, insulin attaches itself to the insulin receptor (INSR) and causes the target tissues to uptake glucose. Tyrosine residues of the insulin receptor substrate IRS-1 or -2 become phosphorylated when insulin binds to its receptor and starts a downstream signaling cascade. Phosphoinositide-dependent kinase-1 (Akt-1 and -2), ribosomal protein S6 kinase beta 1 (S6K1), phosphatidylinositol 3-kinase (PI3K), protein kinase C (PKC), and mammalian target of rapamycin (mTOR) are the next in line. Following this, there will be a greater translocation of glucose to the membrane via glucose transporter 4 (GLUT4) [128]. By controlling the INSR and IRS, several miRNAs have been shown to contribute to insulin resistance. IRS-1 is targeted by miR-96 and miR-126, miR-144 and INSR, and IRS-2 by miR-33, miR-7, miR-27 and miR-13a [128,135]. miR-135 also inhibits the insulin-induced phosphorylation of PI3K/AKT and adversely regulates IRS-2 expression [135]. miR-378 inhibits the production of p110α, the catalytic subunit of phosphoinositide 3-kinase (PI3K). Insulin resistance is caused by the persistent overexpression of miR-378 because PI3K is a crucial transcriptional regulator of insulin [136]. In addition, insulin resistance results from the inhibition of regulatory component p85α by miR-320, which degrades insulin-PI3K signaling [128]. Through its targeting of the oxysterol-binding protein-related protein (ORP)-8, obesity-induced overexpression of miR-143 also contributes to insulin resistance. This causes the suppression of insulin-induced PI3K–AKT pathway activation [136]. The exaggerated expression of miR-29 and miR-153 can also cause insulin resistance by disrupting the AKT phosphorylation pathway [128].

The skeletal muscle, adipose tissue and liver are among the target tissues where miRNA dysregulation also results in insulin resistance. miR-122 controls both the insulin signaling pathway and glucose metabolism in hepatic insulin resistance. By dephosphorylating tyrosine residues in IRS and INSR, a decrease in miR-122 inhibits the action of hepatic insulin [137]. Nguyen et al. demonstrated the crucial role that saturated fatty acid inducible miR-183-5p plays in the pathophysiology of hepatic insulin resistance and the control of the insulin signaling pathway [138]. The overexpression of miR-27a/b inhibits hepatic glucose production and increases hyperglycaemia via targeting fork head box protein O1 (FOXO1) [139]. The overexpression of miR-144, miR-33, miR-148a and miR-128-1, and the inhibition of miR-30c, miR-224, and miR-520d, are associated with an increase in low-density and reduction in high-density lipoprotein (HDL) production and LDL clearance in hepatocytes [117]. Due to the inhibition of insulin sensitivity and glucose uptake in hepatocytes, an obesity-induced reduction in miR-141-3p can result in insulin resistance [133]. In addition, alterations in miR-125a-5p, miR-21, miR-802, miR-592, miR-29 and miR-543 induce hepatic insulin resistance through various mechanisms [137]. In adipose tissue insulin resistance, it was found that increased levels of a miR-29 family (a, b, and c) negatively affect glucose uptake via lowering GLUT4 expression. Caveolin-1 is the target of miR-107 and miR-103, and it has been discovered that two miRNAs play a critical role in controlling insulin sensitivity. Insulin sensitivity and signaling were improved, fat cell size was reduced, and caveolin-1 expression increased when these two miRNAs were inhibited. Decreased miR-330-5p expression is connected to the emergence of insulin resistance, as this miRNA has an effect on T cell immunoglobulin expression [137]. By inhibiting KLF-15, miR-133a-1, miR-133a-2, and miR-133b reduce the uptake of glucose mediated by insulin in skeletal muscle and adipocytes. Consequently, this implies that miR-133 regulates the pathophysiology of insulin resistance [128]. Decreased miR-182 expression affects enzyme activation and phosphorylation, while miR-16 overexpression plays a role in reducing insulin sensitivity by reducing protein synthesis in muscles. Lastly, it was found that Rock-1 expression levels are inhibited by the overexpression of miR-135, miR-214, and miR-202, which leads to insulin signaling and insulin resistance [137].

**Figure 2:**
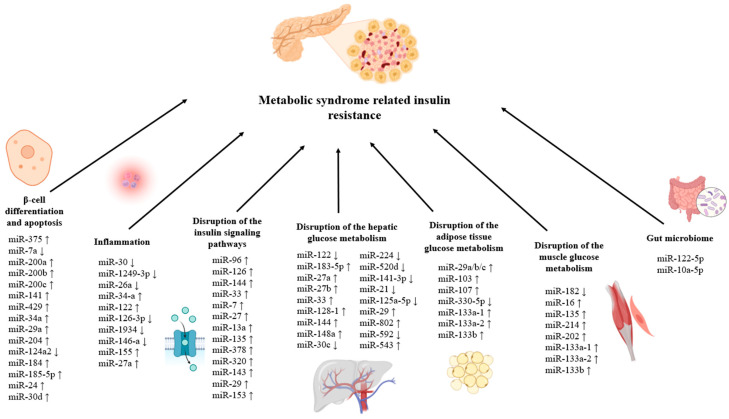
Effects of microRNAs on insulin resistance in MetS. (↓) shows that the expression of the relevant miRNA decreases, (↑) shows that the expression of the relevant miRNA increases.

The gut microbiota–miR crosstalk is also important in maintaining the insulin signaling pathway and glucose homeostasis. It was demonstrated that the expression of miR-122-5p was adversely connected with specific gut bacteria, such as *Phascolarctobacterium faecium* and *Bacteroides uniformis*. Since miR-122-5p is essential for controlling glucose metabolism, these results imply that alterations in the gut microbiome may influence glucose homeostasis and insulin resistance through modulation of this microRNA [140]. Moreover, oral miR-10a-5p therapy lowers insulin resistance brought on by a high-fat diet via modifying the circadian cycle of intestinal *Lachnospiraceae* and its metabolite butyric acid [141]. The effects of microRNAs on insulin resistance in MetS are given Figure 2.

In conclusion, circulating miRNAs play a crucial role in the regulation of insulin signaling and glucose metabolism, making them key contributors to the development and progression of insulin resistance. They can change gene expression in tissues that are susceptible to insulin, including muscle, adipose tissue, and the liver. However, current research is still lacking in several key aspects, despite increased interest in circulating miRNAs as possible biomarkers and therapeutic targets for insulin resistance in MetS. Future investigations should focus on larger, well-characterized studies with standardized protocols, investigate the mechanistic involvement of circulating miRNAs in insulin signaling pathways, and evaluate how therapeutic interventions affect the dynamic changes in circulating miRNA profiles.

Xeno-miRNAs could enter circulatory systems and organs, where they may influence health and diseases. It was indicated that food intake modifies the inflammatory conditions and redox homeostasis that underline pathological processes in MetS and MetS-related symptoms via delivering xeno-miRNAs. Nonetheless, their absorption, stability, availability, and epigenetic functions are fascinating and debated topics nowadays, but there is scarce information on the physiological and possible adverse consequences of xeno-miRNAs [36]. miRNA-148a in bovine milk appears to encourage β-cell dedifferentiation to the immature mTORC1-high/AMPK-low phenotype, which is characterized by functional deficiencies in insulin production, increased endoplasmic reticulum stress caused by mTORC1, decreased early β-cell apoptosis, and autophagy. Boiling, ultra-heat treatment, and bacterial fermentation deactivate milk’s miRNAs. This raises the suspicion that the continuous consumption of pasteurized milk may cause diabetes via miR-148a [63]. Similarly, milk-derived miR-21 disrupts the homeostasis of α-synuclein, leading to its overexpression and aggregation. This can have harmful consequences on pancreatic β-cells, perhaps contributing to the pathophysiology of T2DM [36]. miRNAs are a significant component of breast milk. The presence of stem cells and breast milk exosome-derived miRNAs is responsible for the highest concentration of miRNAs in breast milk. Second, breast milk miRNAs are extremely resilient to extreme circumstances like UV light, RNase digestion, low pH, high temperatures, repeated freeze/thaw cycles and pasteurization. Thirdly, only a minor portion of miRNA comes from the circulation of maternal blood; the majority comes from mammary epithelial cells. As a result, a variety of organ-derived miRNAs, including pancreatic, liver and hematopoietic-derived miRNAs, are given to breastfed infants. Additionally, xeno-miRNAs have been discovered in breast milk, proving that miRNAs are not just produced endogenously (by the lactation glands or by the mother’s blood circulation) [142]. Some miRNAs abundant in breast milk are essential for immune system-related pathways: miR-155 regulates the maturation of T and B cells and the innate immune response, miR-92s are responsible for T- and B-cell and monocyte development, miR-181a and miR-181b regulate B-cell differentiation, miR-17 and miR-125b inhibit tumor necrosis factor-alpha production, miR-146b negatively regulates innate immunity, and miR-223 regulates the activation and proliferation of neutrophils [143].

When evaluated in terms of plant-based xeno-miRNAs, it was noted that rice aleurone-derived hvu-miR-168-3p decreased blood glucose levels and enhanced glucose transporter 1 (GLUT1) expression [36]. In another study, it was shown that carrot-derived miRNAs target genes’ regulating pathways (insulin signaling pathway, AMPK signaling pathway, MAPK signaling pathway, TGF-beta signaling pathway, etc.) associated with MetS. It was concluded that the management of obesity and MetS could benefit from the development of curative and preventative methods based on foods that are enriched with these miRNAs [144]. Diez-Sainz et al. have shown that miR162, miR159, and miR156e were found in edible plants in high abundance, and miR159’s projected target genes were associated with the insulin signaling system, the negative control of insulin production involved in the physiological response to glucose stimulation, and the positive regulation of fatty acid beta-oxidation and glucose metabolism. However, these miRNAs were not detected in serum, and their interactions with enterocytes and the gut microbiome are assumed to be the cause of their potential effects [34]. Another study showed that miR-482c-5p and miR-482f from plant-based foods such as fruits and vegetables, grains, legumes, and oils can increase the expression of anti-inflammatory genes in macrophages [145]. Anti-inflammatory activity is crucial in preventing the development and progression of many metabolic conditions. However, although these miRNAs are resistant to destruction during the digestive process, they cannot be absorbed. For this reason, they were not detected in serum and are only effective at the intestinal level [145]. Similarly, it has been shown that dry nut-derived miR159a and miR156c can attenuate the Tnfrsf1a protein and TNF-α signaling pathway in adipose cells, thus effectively suppressing inflammation. It has been noted that these xeno-miRNAs could be crucial in the management of metabolic conditions associated with inflammation, such as insulin resistance [146]. It is also anticipated that the target gene of miR-5781 derived from soybeans is IL-17A, that of miR-4996 is IL-10, and that of miR-5671a is IL-33; the target of miR-164a derived from hami melon is IL-16; that of miR-398b derived from oranges is IL-1α; that of miR-1078 derived from ginger is IL-6; and that of miR-4995 derived from tomatoes is IL-5 [69]. However, new approaches and nanocarriers are needed to extend the bioavailability of plant-derived xeno-miRNAs beyond the intestine [34].

miRNAs can have their tissue expression and plasma levels altered by food, nutrition, diet, or non-nutrient substances. Although studies on xeno-miRNAs are limited [34,63,143,144,145,146], the impact of nutrition on the expression of miRNAs has been studied more broadly. When the effects of foods on miRNAs were evaluated, it was stated that milk consumption causes a rise in the levels of miR-200c and miR-29b, which are linked to insulin resistance [23]. According to clinical data from a cohort of women with overweight and insulin resistance, blood orange juice consumption (500 mL per day) for four weeks resulted in decreased let-7f-5p and miR-126-3p levels in peripheral blood mononuclear cells and increased miR-144-3p, miR-424-5p, miR-144-3p, and miR-130b-3p. The increase in the mentioned miRNAs resulted in the decreased expression of NF-κB and IL-6 [147]. In another study, healthy participants who had 500 mL of orange juice showed an immediately enhanced expression of miR-375 and decreased hyperglycemia brought on by a high-calorie meal [148]. In randomized crossover research including 49 prediabetic patients, Hernández-Alonso et al. found that two weeks of pistachio consumption (57 g/day) decreased miR-375 and miR-192 expressions [149]. One more study by Rodriguez-Mateos et al. found that eating wild blueberries affected the expression of miRNAs linked to the insulin signaling pathway in healthy males. The researchers demonstrated that eating wild blueberries 11 g twice a day decreased miR-30c-5p and miR-126-5p expressions [150]. Mir-let7a was also lower in patients with MetS who drank at least one sweetened beverage, ate at least three pieces of fruit each day, and ate less red meat than white meat [151].

When the effects of nutrients on mRNAs were evaluated, palmitic acid facilitated the development of insulin resistance in hepatic cells by activating the PI3K-AKT pathway [36]. By boosting the expression of miR-96-5p and miR-27a-3p, quercetin and catechin epigallocatechin gallate reduced insulin resistance by lowering the transcription of gluconeogenic enzymes and glucose synthesis [36]. Polyunsaturated fatty acid (PUFA) therapy also resulted in the downregulation of miRNAs linked to inflammation, including miR-21, miR-125a, miR-155, miR-146a and miR-146b [36]. The HDL fraction had a large amount of miR-155, and smoking increased miR-155 expression. The miR-155 level in the HDL_3_ fraction was reduced by 92% in the smoking group, following 8 weeks of vitamin C supplementation (1250 mg/day). This suggests that a high dose of vitamin C can enhance the anti-inflammatory effect by reducing inflammatory miRNA [152]. However, in 21 prediabetic participants, taking 2000 units of cholecalciferol (vitamin D) every day for four months decreased the plasma levels of miR-7 and miR-192 while raising levels of miR-152 [153]. When the impacts of diets on the expressions of miRNA were assessed in a study, the effects of two different dietary strategies for weight loss on miRNA expression in MetS were evaluated. After 8 weeks, it was discovered that 49 miRNAs have varied expression, with 35 associated with the control diet (based on American Heart Association recommendations) and 14 with the RESMENA diet (Mediterranean diet with high meal frequency). Notably, significant expression changes were observed in miR-214, miR-410, miR-190, and miR-637 as a result of the weight-loss intervention [154]. A pilot study reported a decrease in two plasma miRNAs (hsa-miR-26a-5p and hsa-miR-126-3p) that have been shown to be associated with diabetes in a traditional Korean diet group characterized by low red meat, moderate to high fish and legumes, and high fiber and vegetables levels [155]. The regulation of several miRNAs linked to MetS may also be influenced by the Mediterranean diet [156]. It was found that there is a correlation between increased serum levels of miR-590 and Mediterranean diet adherence [151].

In conclusion, xeno-miRNAs can affect fasting glucose and insulin resistance through a range of pathways. Nonetheless, their absorption, stability, availability, and epigenetic functions are fascinating and debated topics nowadays, but there is scarce information on the physiological and possible adverse consequences of xeno-miRNAs [37]. Standardized techniques are required to identify and measure the xeno-miRNAs in target tissues and circulation. Also, studies on this subject are quite limited. Further research is required to completely clarify the mechanisms by which miRNAs affect impaired fasting glucose, to examine their clinical applications in diagnosis and treatment, and to investigate the nutritional sources, metabolism, and mechanisms of xeno-miRNAs.

### 3.2. Abdominal Obesity

**Figure 3:**
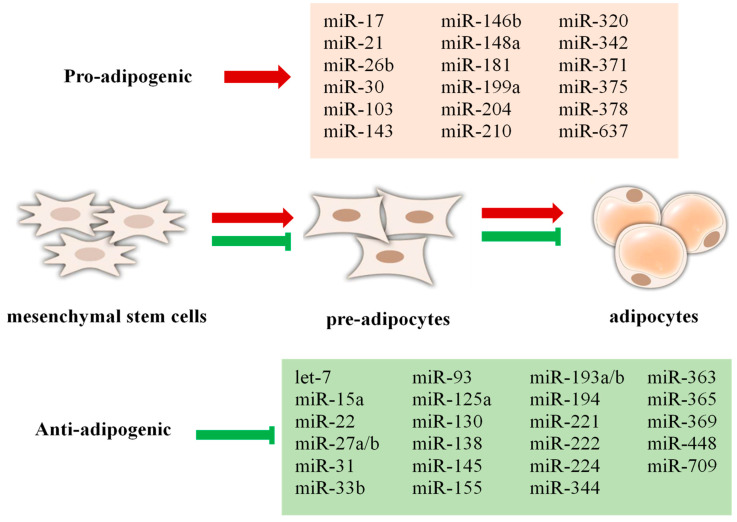
Some miRNAs that promote or inhibit adipogenesis weight increase. Red arrow indicates promote, green arrow indicates inhibit.

Being a major component of MetS, obesity is a complex health problem that occurs as a result of an abnormal increase in fat mass in the body, causing endocrine, metabolic, and behavioral changes [157]. Environmental, microbiological, epigenetic, and genetic risk factors all contribute to obesity. Obesity and associated metabolic disorders arise when epigenetic mechanisms—such as DNA methylation, histone changes, and miRNA-mediated processes—are disrupted [158]. A well-known pathogenic physiological state that involves modifications in different miRNA expressions is obesity. The abnormal expression of miRNAs, which are effective in regulating adipose tissue metabolism, contributes to the emergence of obesity [159]. Exosomal miRNAs in the circulation are mostly derived from adipose tissue, and different fat depots release different exosomal miRNAs into the blood [160]. miRNA genes are associated with brown fat cell differentiation, which plays a role in obesity [161]. Some miRNAs that promote or inhibit adipogenesis are shown in Figure 3 [162,163].

The development of obesity involves the interplay of multiple biological pathways. miRNAs have been shown to modulate essential metabolic pathways, including adipogenesis, lipogenesis, glycolysis, gluconeogenesis, thermogenesis, and cytokine signaling [164]. A diet with high fat is a major factor contributing to the development of obesity, leading to increased lipogenesis, fat accumulation, and overall body weight gain. miRNA-143 expression plays a role in the regulation of lipogenesis by increasing the glycoprotein nonmetastatic melanoma protein B (GPNMB), which the liver secretes, and this encourages white adipose tissue to produce lipogenesis [164,165]. In a study, miR-145 was shown to attenuate lipolytic activity in white adipose tissue by directly suppressing the expression of key lipolysis-promoting factors, including FoxO1 and abhydrolase domain containing 5 (ABHD5/Cgi58) [166]. It has been shown that miR-345-5p, which is negatively associated with obesity, is decreased when focusing on vascular endothelial growth factors during adipogenic differentiation. miR-345-5p upregulation contributes to decreased fat storage in adipose tissue, and plays a key role in controlling gene expression related to lipid metabolism, including lipogenesis, fatty acid biosynthesis, and transport mechanisms [167].

Adipogenesis can be defined as the process of increased adipocyte formation and differentiation into mature adipocytes [168]. In one study, miR-27a-3p was shown to be a critical regulator of human adipogenesis. This miRNA promoted fat cell formation by increasing the expression of genes that promote adipocyte differentiation. These results imply a potential regulatory function of miR-27a-3p in the adipogenic differentiation process [169]. In a study conducted in bone marrow mesenchymal stem cells, miR-422a and miR-483-5p were shown to promote the adipogenesis process. These miRNAs suppressed the expression of the Methyl-CpG binding protein 2 gene, leading to an increase in adipocyte markers (adiponectin, leptin, FABP4). In addition, miR-422a was found to inhibit osteogenesis markers and was specifically increased during adipogenesis. miR-422a appears to exert a significant influence on the molecular pathways governing adipocyte formation [170].

Brown adipose tissue, known for its involvement in body fat regulation and energy homeostasis, contributes to reduced adiposity primarily through the enhancement of thermogenic activity. By altering thermogenic energy expenditure and brown adipogenesis, some miRNAs contribute to obesity [171,172]. Brown adipocyte differentiation is negatively regulated by miR-133, but brown adipogenesis is positively regulated by miR-196a. The overexpression of miR-196a in adipose tissue prevents obesity by increasing brown adipose tissue activity and energy expenditure [173].

The first miRNA linked to metabolic disorders in humans is miR-122, which is found in large quantities in the liver. It contributes to the control of lipid metabolism and cholesterol [174]. Increased miRNA-122 levels reduce brown adipose tissue activity [173]. Furthermore, it has been demonstrated that there is a positive correlation between BMI and the level of miR-122 in the blood [174]. A prospective population-based study identified elevated circulating levels of miR-122 as a significant predictor of the development of MetS in the general population [175]. Circulating miR-122 expression was shown to be considerably higher in patients with MetS than in healthy controls in a study of those with the diagnosis, and there was a positive association between miR-122 levels and BMI. These results imply that miR-122 could be a viable biomarker candidate for MetS diagnosis [176].

Numerous circulating microRNAs, such as miR-101a, miR-16, miR-186, miR-200c, miR-21, miR-299, miR-30c-2, miR-467b, and miR-877, have been linked to the regulation of adiposity [23]. In a study exploring the associations between circulating miRNAs and parameters such as obesity, the distribution of adipose tissue, and total fat accumulation, twelve miRNAs were identified as significantly correlated with these phenotypes. Among them, eight miRNAs—including miR-149-3p, miR-193a-5p, miR-4478, miR-7107-5p, miR-6088, miR-6799-5p, miR-6803-5p, and miR-6821-5p—showed positive associations, whereas four miRNAs—miR-4433b-5p, miR-345-5p, miR-145-5p, and miR-3937—were inversely related to adiposity measures [177].

Heianza et al. reported that adipose tissue-related miRNA-99/100s control PPARγ expression, adipose tissue inflammation, lipid storage, and other critical metabolic factors, hence modulating adiposity, weight gain, and liver steatosis. They reported that reductions in the expression of miR-99-5p/100-5p in circulation induced by dietary therapy and physical activity intervention were linked to both ectopic fat formation and better body fat distribution [178].

Adipocyte-derived adipokines are key regulators of various metabolic processes, including energy homeostasis, inflammatory responses, and the pathophysiology of obesity. There is an interaction between adipokines and miRNAs in maintaining metabolic homeostasis [171]. miRNAs are key regulators of adipokine production and release in adipose tissue. Conversely, certain adipokines have also been demonstrated to affect particular miRNAs’ expression profiles, indicating a bidirectional regulatory relationship between miRNAs and adipokine signaling [30]. The elevated expression of miR-27b and miR-145 in obese patients has been associated with increased leptin receptor expression and activity, implying a possible regulatory role in leptin-mediated metabolic processes [179]. A study found that leptin and resistin significantly reduced miR-143 expression in human preadipocytes [180]. In a study conducted on morbidly obese adolescents, reduced levels of miR-15a, miR-146a, miR-423-5p, miR-520c-3p, and miR-532-5p and elevated levels of miR-130, miR-140-5p, miR-142-3p, miR-143, and miR-222 were detected. These circulating miRNAs were strongly associated with waist–height ratio, BMI, adiponectin, leptin, leptin-adiponectin ratio, fasting blood glucose, C-peptide, insulin, HOMA-IR, and circulating plasma lipids (triglyceride, LDL-C, and HDL-C). It has been demonstrated that adipokines and factors related to MetS may change through the regulation of miRNA expression [174].

Dietary habits and endogenous miRNA regulation are tightly associated. In particular, the energy content of the diet and fat intake have been implicated as key factors affecting miRNA levels [53]. In a systematic review, energy intake has been reported to be an important regulating factor in the human miRNA profile. Changes in endogenous miRNA levels are caused by variations in dietary energy. The length of the dietary intervention and the nutritional circumstances may affect these results. In addition, it has been suggested that these miRNAs have an effect on metabolic pathways and alter the development process of metabolic diseases. It has been stated that miR-22 controls metabolic homeostasis, and treatment for metabolic disorders like obesity and hepatic steatosis may benefit from suppressing this miRNA [53]. A meta-analysis examining extracellular miRNAs in the context of weight loss revealed that circulating levels of miR-223, miR-126, and miR-26a significantly increased post-weight reduction, whereas miR-142 levels showed a marked decrease. In contrast, miR-122, miR-140, miR-146, and miR-221 exhibited no notable changes [181]. After the energy-restricted diet, miR-193a-5p and miR-122-5p levels decreased with weight loss, while miR-126a-3p and miR-222-3p levels increased [182].

The Mediterranean diet, which possesses antioxidant, anti-inflammatory, and anti-atherosclerotic properties, is effective in regulating various miRNAs associated with MetS. In a study conducted in morbidly obese people, higher serum miR-590 levels were were linked to increased Mediterranean diet adherence. A healthier miRNA profile in circulation was linked to greater adherence to the Mediterranean diet, while high-calorie diet consumption was linked to elevated levels of miRNAs involved in the onset and progression of obesity [151].

The inclusion of specific polyphenols or plant-derived bioactive compounds in the diet leads to notable alterations in peripheral tissue miRNA expression, suggesting a mechanistic link between dietary components and gene regulation. In particular, these compounds affected the miRNA dysregulation that would lead to obesity, permitting weight loss and adipogenesis inhibition [183]. In an in vivo study with curcumin supplementation, curcumin inhibited adipogenic differentiation by suppressing miR-17-5p expression in white adipose tissue [184]. In a study including people with MetS who received daily resveratrol (150 mg/day resveratrol and 250 mg/day δ-tocotrienol) supplement as a mixture for six months, improvements were observed in MetS characteristics, including hypertension, dyslipidemia, impaired fasting glucose, and central obesity. Via the examination of serum miRNA levels of individuals, it was shown that resveratrol supplementation significantly reduced the expression of miR-122 and, statistically, considerably decreased the expression of miR-130b and miR-221-5p. Among the miRNAs whose expressions changed, miR130b contributed to the prevention of central obesity in particular by suppressing PPAR-γ, which contributes to the development of adipocytes and adipogenesis in adipose tissue [185]. Green tea, rich in flavonoids and polyphenolic constituents, exerts various health-promoting effects, including the enhancement of redox balance, the attenuation of inflammatory responses, and improved glucose and lipid metabolism [186]. One study evaluated the potential of green tea supplementation given to obese women to alter miRNA levels by reducing markers of oxidative stress and inflammation induced by a high-fat and high-saturated-fat meal. Green tea reduced the levels of 62 miRNAs that are activated after a fatty meal and prevented these molecules from affecting cellular signaling pathways such as TGF-beta, methyltransferase 1, S6 kinase, and bone morphogenetic proteins [187]. Selenium protects against oxidative stress through selenoproteins, and is therefore important in reducing the occurrence of chronic diseases. In a study conducted to determine the effects of Brazil nut intake on circulating miRNAs in obese women with and without MetS, one Brazil nut per day (approximately 1261 μg Selenium) was given for 2 months. Circulating expression levels of miR-454-3p and miR-584-5p rose in obese women at the end of the research, whereas miR-375 levels dramatically dropped in women with MetS. Furthermore, a relationship has been shown between calcium homeostasis, vitamin D metabolism, and selenium intake [188].

It has been stated that miRNAs can respond to various nutritional interventions and can be regulated by changes in diet and lifestyle factors, thus correcting metabolic disorders [163]. Table 2 summarizes the changes in miRNAs according to nutritional interventions, diet, and lifestyle factors.

According to recent research, dietary intake containing xeno-miRNAs modulates individual miRNA profiles and alters inflammation and redox homeostasis, which are the foundations of diseases like insulin resistance, type 2 diabetes, cancer, and MetS [195,196]. Despite growing interest, the role of food-derived xeno-miRNAs in human health and disease has not yet been clearly elucidated, and their clinical implications remain largely uncertain [36].

One of the important sources of miRNAs is breast milk [197]. miR-22-3p, miR-200a/c-3p, miR-148a-3p, miR-30a/d-5p, miR-146b-5p, and members of the let-7 family have been identified in the cellular, lipid, and skim milk components of human breast milk, which represents the initial source of nutrition in the neonatal period [198]. There are some factors that affect these miRNA expressions, both maternal and milk-related. Factors related to the mother include pregnancy nutrition, pregnancy body weight, stress status during pregnancy, time of birth of the baby, delivery method, mother’s nutrition, mother’s body weight and chronic disease conditions of the mother, while factors related to milk include breast milk storage conditions and heat application to breast milk [199]. A study on breast milk sought to determine how maternal obesity affected specific miRNAs (miR-148a, miR-30b, miR-29a, miR-29b, miR-let-7a, and miR-32) involved in glucose metabolism and adipogenesis, as well as how these miRNAs related to measurements of infant body composition during the initial six months after birth. In this study by Shah et al., it was reported that miRNA-30b and miRNA-148a were abundant in breast milk exosomes, and the amounts of these miRNAs were affected by the mother’s body weight and breastfeeding duration. At one month, miR-30b was favorably correlated with infant body weight, body fat percentage, and fat mass, but miR-148a was negatively correlated with infant body weight, fat mass, and lean mass. A certain miRNA content in human milk was inversely correlated with maternal obesity. It has been reported that miRNA 148a and 30b may be significant indicators of early infancy fat accumulation and baby growth [197]. Milk-derived miR-148a and miR-30b have been associated with adipogenic effects, suggesting a relationship between the prevalence of obesity and milk consumption [91].

As a result, miRNAs play important roles in the pathophysiology of obesity, and the potential roles of these molecules as both therapeutic agents and diagnostic biomarkers have been revealed. miRNAs function in many biological processes associated with obesity, such as energy metabolism, adipose tissue homeostasis, inflammation, insulin resistance, and cellular stress by regulating gene expression at the post-transcriptional level. In this context, miRNAs are considered promising targets in the early diagnosis of obesity-related metabolic complications and in individualized treatment approaches. However, changes in lifestyle, like improving diet, exercising more, and losing weight, can alter miRNA expression profiles, and we still do not fully understand the long-term effects of these changes. The lack of longitudinal studies examining the permanent effects of lifestyle changes applied in obesity treatment on miRNAs limits the sustainability of these molecules as biomarkers and their stability as therapeutic targets. Therefore, future research should focus on prospective studies to evaluate the effects of lifestyle interventions on these molecules in the long term. Thus, the clinical use of miRNAs in obesity diagnosis, follow-up, and treatment will be based on more solid scientific foundations.

### 3.3. High Blood Pressure

One of the main components of the MetS is hypertension, and its complications cause significant increases in morbidity and mortality. The renin–angiotensin–aldosterone system (RAAS), insulin resistance, obesity, catecholamines, oxidative stress, inflammatory mediators, and high fructose and salt intake are all MetS-related factors that lead to the development of hypertension [200]. miRNAs control hypertension through intricate and varied molecular processes [18]. Some miRNAs have been reported to affect blood pressure and the development of hypertension through vascular, renal, and other physiological mechanisms [201]. In total, 30 miRNAs have been identified that are abundant in human microvascular endothelial cells, and are associated with target genes known to be involved in blood pressure regulation or hypertension [202]. Some miRNAs that act on the kidney to regulate hypertension have been shown to include miR-214-3p, miR-195-5p, miR-192, miR-181a, and miR-133a. miR-214-3p reduces arterial pressure by suppressing endothelial nitric oxide synthase. miRNA-195-5p reduces hypertension by inhibiting Na-K-2Cl cotransporter 2 isoform A (NKCC2A) along the thick ascending limb of the loop of Henle. miR-192 targets Na-K-ATPase β1, a transporter; miR-133a acts by targeting angiotensinogen in the proximal tubule. miR-181a regulates blood pressure by affecting the renin–angiotensin–aldosterone system [201].

It has been proposed in recent years that miRNAs may serve as possible biomarkers for numerous pathological conditions, including hypertension [203]. The combination of miR-122-5p, miR-199a-3p, miR 223-3p, and miR-208a-3p has been suggested as a potential marker for the diagnosis of hypertension, and miRNA dysregulation raises the risk of developing hypertension [204].

The expression of miRNA is changed in the context of hypertension. A study has shown that miR-126-3p and miR-182-5p expressions are significantly higher in hypertensive individuals than in normotensive individuals. It has also been emphasized that these miRNAs can be used for the diagnosis of hypertension, and are important for the development of miRNA-based treatments of hypertension [205].

Inflammatory processes, including endothelial dysfunction, vascular inflammation, and reactive oxygen species (ROS) production, are effective in the formation of hypertension [206]. These inflammatory processes are mediated by miRNAs [207]. Exosomal miRNAs affect the balance between proantioxidant and antioxidant components, including superoxide dismutase (SOD) and NAD(P)H oxidase (NOX) enzymes. miR-21 downregulates SOD, which protects against ROS with antioxidants. It has been reported that miR-21 directly binds to SOD3 in the 3′-UTR region to regulate ROS levels, and indirectly stimulates SOD2 expression by suppressing tumor necrosis factor-alpha (TNF-α). In addition, miR-21 reduces ROS levels by increasing cytochrome b expression in mitochondria. It is thought that the ROS balance disrupted in hypertension may be corrected in this way [208]. Likewise, many miRNAs, including miR-155, miR-212, miR-21, miR-19a/b, and miR-20b, mediate inflammatory processes that lead to hypertension [209].

miRNAs have been shown to regulate the expression of blood pressure-associated genes within the RAAS, thereby contributing to the molecular mechanisms underlying the development of hypertension. The RAAS functions as a critical neurohormonal system that maintains blood pressure homeostasis and electrolyte balance through tightly regulated endocrine signaling pathways [209]. It has been shown that exosomal miRNAs can regulate the RAAS pathways, such as the angiotensin-converting enzyme (ACE)/angiotensin II pathway and the ACE2/Mas pathway. It has been demonstrated that the overexpression of miR-155-5p lowers blood pressure and vascular proliferation by directly lowering ACE gene and angiotensin II peptide levels [210]. In one study, miRNA-143/145 was found to reduce ACE1 expression via adenosine monophosphate-activated protein kinase α2 (AMPKα2), thus slowing down the conversion of Angiotensin I to vasoconstrictive Angiotensin II and showing atheroprotective and antihypertensive effects in endothelial cells [211]. Another miRNA with antihypertensive effects, miR-181a, directly reduces renin levels. The excessive activation of the sympathetic nervous system, which is associated with high blood pressure, inhibits miR-181a expression, and thus increases renin activity, leading to increased blood pressure [209,212].

miR-122 has been implicated in the regulation of multiple target genes associated with the renin–angiotensin system, thereby influencing key pathological processes such as hypertension and cardiovascular fibrosis. Furthermore, sirtuin 6 (SIRT6), elabela, growth differentiation factor 15 (GDF15), porimin, and connective tissue growth factor (CTGF) are among the factors that miR-122 controls in relation to cardiovascular function. In addition, miR-122 causes angiotensin II-induced endothelial dysfunction and vascular fibrosis by targeting sirtuin 6 (SIRT6), a negative regulator of the renin-angiotensin system [18]. Angiotensin II plays a crucial part in the pathophysiological increase in blood pressure by stimulating the proliferation of vascular smooth muscle cells through its vasoconstrictive effects [213].

**Figure 4:**
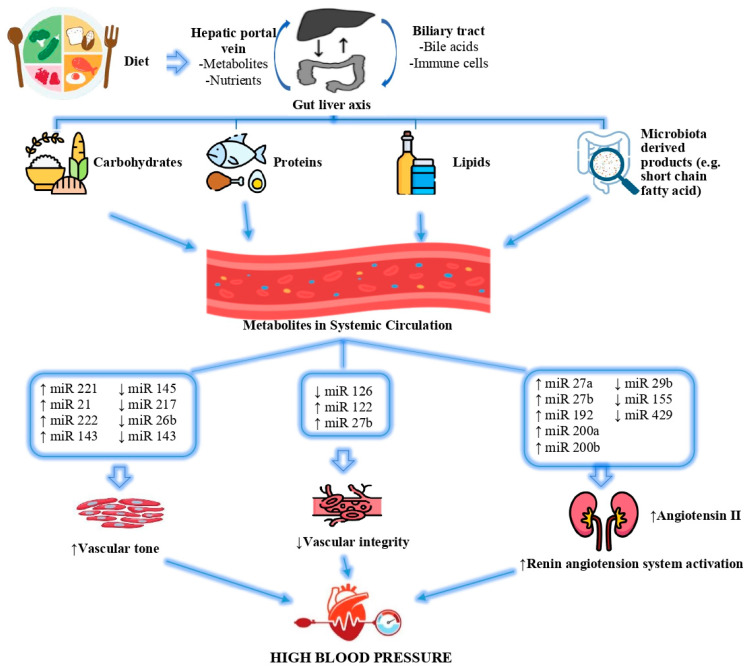
Effects of altered miRNA expressions in response to diet on the development of high blood pressure [215]. (↓) shows that the expression of the relevant miRNA decreases, (↑) shows that the expression of the relevant miRNA increases.

Environmental and genetic factors cause dysbiosis by inducing pathogenic bacteria to multiply and disrupt the intestinal microbiota. It is suggested that the changing microbiome composition with intestinal dysbiosis affects the pathogenesis of essential hypertension [214]. miRNA expression may be impacted downstream by dietary disruptions in the gut–liver axis, as evidenced by the study of nutritional epigenetics. Both macronutrients and micronutrients have been reported to influence regulatory miRNAs, thereby altering multiple cellular processes that contribute to hypertension and its comorbidities [215]. The effects of altered miRNA expressions in response to diet on the development of high blood pressure are shown in Figure 4.

Excessive dietary sodium consumption is a major environmental determinant of blood pressure regulation, and is strongly associated with an increased risk of cardiovascular disease. Although numerous complex and interrelated physiological pathways have been implicated in the salt–blood pressure relationship, many of these underlying mechanisms remain incompletely understood [216,217]. Studies have shown that changes in miRNA expression play a role in the pathological events underlying hypertension [218,219]. In one study, reducing dietary sodium intake increased circulating levels of miR-143-3p. The increased expression of miR-143-3p has been shown to reduce systolic blood pressure and improve arterial stiffness and skin capillary density [220]. TNF-α, produced by kidney epithelial cells and known for its proinflammatory effects, has been shown to reduce increases in blood pressure in response to high sodium intake by inhibiting NKCC2A [221]. In fact, another study by the same authors determined that TNF-α, which increases with high sodium intake, induces miRNA-195a-5p, and miRNA-195a-5p, and in turn achieves this reduction in blood pressure through a mechanism that regulates NKCC2A mRNA [222].

Unlike the well-known effects of excessive sodium intake, insufficient potassium consumption also contributes to elevated blood pressure, and is recognized as an independent risk factor. Blood pressure regulation exhibits sensitivity not only to sodium but also to potassium, with this trait influenced by both genetic and environmental factors [223]. In a dietary intervention study examining sodium and potassium intake, researchers identified notable associations between genetic variations in specific miRNAs and individuals’ blood pressure responses to these dietary changes. For instance, polymorphisms such as rs115254818 in miR-26b-3p, rs11191676 and rs2292807 in miR-1307-5p, and rs6601178 in miR-4638-3p were linked to increased blood pressure following high sodium intake. Meanwhile, some of these same variants—particularly in miR-26b-3p and miR-1307-5p—were associated with blood pressure reduction after potassium supplementation. Moreover, the study tracked participants over a 14-year period, and found that miRNA-related genetic polymorphisms were significantly correlated with both long-term blood pressure trajectories and the risk of developing hypertension [224].

Fructose, an independent risk factor for hypertension, affects the expressions of some miRNAs that play a role in the pathogenesis of hypertension. While the expressions of miR-19b and miR-101a, which have anti-atherogenic properties, are suppressed after a high-fructose diet, the expression of miR-145a is increased. In addition, high fructose consumption changes blood pressure by affecting miRNAs that affect the renin–angiotensin–aldosterone system [215].

In conclusion, it has been demonstrated that miRNAs may influence the onset of hypertension by controlling the renin–angiotensin–aldosterone system’s constituent parts, peripheral resistance-inducing processes like inflammation, and modifications to the structural and functional characteristics of vascular smooth muscle and endothelial cells. miRNA expression is altered in hypertension. Excessive dietary salt consumption, a modifiable factor, alters blood pressure by affecting circulating miRNA expressions. More comprehensive, prospective human studies are needed to determine how miRNA expression changes in patients with hypertension, and to clarify the long-term effects of miRNAs on hypertension and their potential use in clinical applications.

### 3.4. Dyslipidaemia

**Figure 5:**
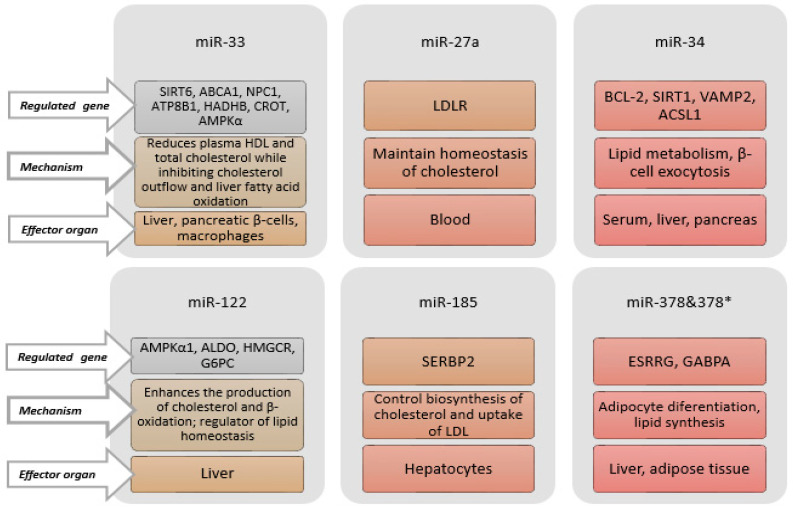
Some miRNAs related to lipid metabolism, regulated genes, mechanisms, and effector organs. ABCA1: ATP-binding cassette subfamily A member 1. ACSL1: Long-chain acyl-CoA synthetase 1. ALDO: Aldolaz. AMPKα: AMP-activated protein kinase alpha. ATP8B1: ATPase phospholipid transporting 8B1. BCL2: B-cell lymphoma gene-2. CROT: Carnitine o-octanoyltransferase. ESRRG: Estrogen Receptor-Related Receptor. GABPA: GA-Binding Protein Transcription Factor Subunit Alpha. G6PC: Glucose-6-Phosphatase Catalytic. HADHB: Hydroxyacyl-Coa Dehydrogenase Trifunctional Multienzyme Complex Subunit Beta. HDL: High-density lipoprotein. HMGCR: 3-Hydroxy-3-Methylglutaryl-CoA Reductase. LDL: Low-density lipoprotein. LDLR: Low-density lipoprotein receptor. NPC1: Niemann–Pick disease, type C1. SERBP2: Sterol Regulatory Element Binding Protein 2. SIRT1: Sirtuin 1. SIRT6: Sirtuin 6. VAMP2: Vesicle-Associated Membrane Protein 2.

Lipid transport, fat cell development, and cholesterol synthesis are all regulated by miRNAs, which in turn can impact lipid metabolism [100,225,226,227]. Because miRNAs affect the expressions of genes that control the metabolism of HDL, LDL, and very-low-density lipoprotein (VLDL), they play a role in maintaining cholesterol homeostasis and lipoprotein metabolism [228]. These miRNAs are emerging as new biomarkers for dyslipidaemia, and play significant roles in lipid metabolism by controlling the expressions of genes related to lipid metabolism, such as ATP-binding cassette subfamily A member 1 (ABCA1) and LDL receptor (LDLR) [229,230]. Some miRNAs associated with lipid metabolism, the genes they regulate, their mechanisms, and target organs are shown in Figure 5.

Among these, miR-122, miR33, and miR-27a attract attention due to their role in regulating lipid homeostasis genes [231,232,233]. miR-27a controls the metabolism of cholesterol [229], and miR-33a is a possible biomarker for treatment targets due to its responses to dietary interventions [234]. In patients with dyslipidaemia, synbiotic supplementation has been shown to significantly reduce the expression levels of miR-27a and miR-33a [235]. By enhancing reverse cholesterol transport, the downregulation of miR-33a via elevated ABCA1 and ABCG1 gene expression may raise circulating HDL-C levels [236]. Additionally, Simionescu et al. found that miR-33a expression had an important positive connection with total cholesterol (TC), TAG, LDL-C, and apoB-100, and an inverse correlation (albeit not statistically significant) with HDL-C [237]. It has also been shown that the inhibition of miR-27a can increase the expression of the LDLR (a primary pathway for clearance of LDL-C from circulation) gene, and reduce LDLR degradation by decreasing proprotein convertase subtilisin/kexin type 9 (PCSK9) levels [238].

It has been shown that miRNAs in HDL are different in normal and familial hypercholesterolemic individuals, and have potential for use as biomarkers [239]. The best-studied and best-characterized miRNAs among those involved in HDL-C metabolism are members of the miR-33 family, specifically miR-33a and miR-33b. The intronic miRNA known as the miR-33 family is encoded by the sterol regulatory element-binding proteins (SREBP)-2 and SREBP-1 genes, which are found in intron-16 of two protein-coding genes for SREBP [240]. Together with their host genes SREBP-2 and SREBP-1, miR-33a and miR-33b regulate cholesterol efflux and fatty acid metabolism by preserving the equilibrium between the transcriptional induction and transcriptional repression of genes involved in lipid metabolism [241,242]. The main way that miR-33 controls the metabolism of cholesterol is by directly blocking the ABCA1 and ABCG1 transporters [240,241,242]. While the antagonism of miR-33 with antisense oligonucleotides (ASOs) is sufficient to upregulate hepatic ABCA1 and ABCG1 and boost circulating HDL-C, the overexpression of miR-33 downregulates ABCA1 and ABCG1, lowering HDL-C levels in plasma [241,242]. Patients with MetS have higher levels of VLDL and lower levels of serum HDL-C due to elevated levels of miR-33b and the host gene SREBP1C in the liver. MiR-33a/b inhibition raises HDL-C and lowers VLDL plasma levels [243]. Price et al. demonstrated that AMP-activated kinase (Ampkα1), which governs lipid metabolism, is expressed in response to miR-33a. When miR-33a inhibits AMPKα1, intracellular levels of fatty acids and cholesterol may rise. Thus, by promoting the breakdown of fatty acids, the endogenous suppression of miR-33a in human hepatic cells lowers cholesterol levels [244]. Additionally, one study demonstrated that the suppression of miR-33a and miR-33b resulted in higher HDL-C and lower VLDL-C plasma levels. These findings imply that creating miR-33 antagonists could be a viable method of treating dyslipidemia linked to MetS and cardiometabolic diseases [31,243].

The first miRNA linked to metabolic control, miR-122, is positively correlated with LDL-C, and exhibits increased expression in those with MetS [245]. miR-122 contributes to the protection of liver functions by playing a role in maintaining liver cholesterol balance and regulating fatty acid metabolism. It shows these effects by regulating gene expression and affecting regulatory enzymes involved in cholesterol biosynthesis. As a result, LDL, HDL, TC and apolipoprotein B levels decrease [29,233]. In patients with type 2 diabetes, miR-122 has also been found to exhibit a positive and important connection with LDL-C. [233]. Similarly, in T2DM patients with and without coronary artery disease, circulating miR-122 was found to be significantly correlated with LDL-C, TG and TC by Rashed et al. [246]. When comparing individuals with hyperlipidemia to the control group, Gao et al. discovered a positive correlation between miR-122 expression and LDL-C, TG and TC [247]. The significant down-regulation of genes involved in de novo cholesterol synthesis, including squalene epoxidase (SQLE), seven dehydrocholesterol reductase (DHCR7), 3-hydroxy-3-methylglutaryl-CoA reductase (HMGCR), and 3-hydroxy-3-methylglutaryl-CoA synthase 1 (HMGCS1), was observed upon the suppression of miR-122 [248]. In miR-122 suppression settings, a 25–35% reduction in total plasma cholesterol was noted, which is consistent with these findings [245]. Fatty acid and cholesterol synthesis is decreased when miR-122 expression levels for a number of genes, including SREBP, are downregulated, whereas obesity results from overexpression, according to Novák et al. [249]. According to one study, dyslipidemia and elevated levels of miR-122 in the blood were associated positively [175]. In addition, miR-122 has been shown to play an important role in regulating TC and TG levels. It shows this effect by controlling cholesterol biosynthesis in the liver and leading to the release of VLDL. The inhibition of miR-122 has been shown to reduce cholesterol and lipid accumulation in the liver by decreasing the expressions of various genes involved in regulating lipid biosynthesis [244]. A study by Rottiers and Näär showed that one of the significant metabolic roles of miR-122 and miR-33 is maintaining cholesterol levels and lipid biosynthesis [31]. Although miR-33 and 122 still require studies to be fully integrated into clinical practice, applying these two miRNAs in treating lipid disorders is important and promising for the future [250].

By controlling hepatic LDLR expression and VLDL generation, several miRNAs, including miR-148a, miR-128-1, miR-483, miR-520d, miR-224, miR-30c, and miR-122, regulate plasma LDL-C and VLDL-C levels [251]. By raising hepatic LDL receptor and ABCA1 expression, the inhibition of miR-148a lowers LDL-C and raises HDL-C levels (in vivo) [252]. In a systematic review examining the links between MetS components and circulating miRNA, lower levels of miR-363, miR-375, miR-486 and miR-16 were observed in the entire cohort (primarily in men when considered by gender) in association with lower HDL concentrations [25]. These miRNAs have been implicated in bile acid production and secretion, cholesterol efflux, hepatic uptake, and HDL-C biogenesis [253]. In a systematic review, it was reported that the levels of sixteen circulating miRNAs (miR-629-5p, miR-33, miR-375, miR-454-3p, miR-320b, miR-25-5p, miR-1180, miR-103, miR-339-5p, miR-628-3p, miR-29a-3p, miR-122-5p, miR-29b-2-5p, miR-185-5p, miR-19a-3p, miR-886-5p) were associated with HDL-C (p < 0.05) [121]. In another review, it was reported that miR-302, miR-27, miR-148a, miR-34a, miR-10b, miR-128-1, miR-26, miR-758, miR-19b and miR-144 regulate plasma HDL-C levels by directly affecting ABCA1 expression in the liver or macrophages [228]. miR-34a is known to be one of the primary regulators of hepatic lipid homeostasis [31].

Overweight/obese persons with hypertension have higher levels of miR-605 and miR-623; there have been reports of negative correlations between miR-623 and HDL-c levels and positive correlations between miR-605 and TC and LDL-C levels [254]. According to one study, dyslipidemia is linked to plasma markers that indicate diabetes dyslipidemia, and miRNAs including miR-143, miR-122, miR-132, miR-218, miR-21, and miR-155 are involved in both the up- and the down-regulation of these indicators [255]. Additionally, there is a positive correlation between plasma LDL-C and triglyceride levels and let-7 family miRNA levels [256]. Furthermore, it has been documented that people with insulin resistance and/or hyperlipidemia express more miR-33b, miR-146a, miR-10a, miR-125a, miR-33a and miR-21. Further, it has been stated that these microRNAs show a positive correlation with TC, TG and LDL-C levels in individuals with hyperglycemia and hyperlipidemia [5,237]. Table 3 summarizes the correlation between specific miRNAs and lipid biomarkers in populations with MetS components.

The discovery of exogenous plant miR-168a, which has been proposed as a new biomarker for its likely functional role in the metabolism of LDL-C, enables the identification of food-derived xeno-miRs in disease etiology [88,261]. Studies have shown that xeno-miRNAs can perform cross-kingdom roles [88,262,263]. For instance, it has been demonstrated that rice’s miR-168a controls the expression of genes in mammals upon dietary intake [88,263]. Following rice consumption, in vitro and in vivo studies showed that miR168a may bind to the mRNA of the human/mouse low-density lipoprotein receptor adaptor protein 1 (LDLRAP1), suppress the liver’s expression of LDLRAP1, and thus reduce the elimination of LDL from mouse plasma [88,89]. Compared to control mice, rice-fed mice showed higher levels of circulating LDL-C and a greater buildup of miR168a in the liver. Consequently, it was shown that miR168a can decrease LDL clearance in the bloodstream by directly targeting LDLRAP1 in the liver. Exogenous plant miRNAs from a variety of sources have been shown to accumulate in particular tissues, enter the circulatory system from the gastrointestinal tract, and have significant biological effects [88]. In contrast, Dickinson and colleagues reported that they could not detect miR168a in the serum of mice after rice consumption, and did not observe any changes in LDLRAP1 expression levels in the liver. However, they stated that this result may have been due to the experimental methods and applications employed [264].

Because of their structural resemblance to cholesterol, phytosterols work in a similar way. The absorption of cholesterol is decreased when consumed alongside plant-based diets [265]. The daily consumption of a nutraceutical combination including 400 mg phytosterols, 100 mg bergamot extract, 20 mg olive extract, and 52 μM vitamin K2 for up to 12 weeks did not change the serum lipid profile or inflammation-associated microRNAs and biomarkers, according to a study on hypercholesterolemia [266]. Diet-induced dyslipidemia can be avoided by downregulating miRNA-96 and consuming a high-fat, high-carb meal together with foods that include a blend of polyphenolic flavonoids, such as orange juice and grape seed extract [267].

In conclusion, by controlling important genes involved in lipid synthesis, transport, and degradation, miRNAs may have an impact on lipid metabolism. Circulating miRNAs may be potential biomarkers that can be used to assess changes in lipid profiles and monitor responses. Some dietary xeno-miRNAs may also affect lipid metabolism, but studies investigating the effects of dietary xeno-miRNAs on dyslipidemia are quite limited, and current findings are contradictory. Further studies are needed to elucidate the dietary sources and mechanisms of xeno-miRNAs in lipid homeostasis.

## 4. Conclusions and Future Perspectives

miRNAs and diet-derived xeno-miRNAs have received increasing attention for their potential effects on MetS and related pathological processes. As we know, a single miRNA can affect the expressions of several genes, and multiple miRNAs can affect the expression of a single gene. MiRNAs may, therefore, be crucial biomarkers for the prompt diagnosis, prognosis, and management of metabolic disorders. This review offers a current, thorough analysis of the important and contentious role that circulating miRNA and xeno-miRNA activities play in regulating chronic processes linked to MetS and its constituent parts.

miRNAs have been shown to affect T2DM by influencing β-cell differentiation, GLUT4 expression, and insulin sensitivity; obesity by affecting adipogenesis, lipolysis, and energy metabolism; hypertension through their regulatory effects on the renin–angiotensin system and vascular tone; and dyslipidemia by modulating cholesterol and lipoprotein metabolism (HDL, LDL, and VLDL levels). With our current review, it can be concluded that there are significant relationships between miRNA levels and abdominal obesity, insulin resistance, hypertension, and plasma high LDL-C and low HDL-C concentrations. With our current review, it is possible to say that there are important relationships between miRNA levels and abdominal obesity, insulin resistance, hypertension, and plasma high LDL-C and low HDL-C concentrations.

Nutrition and diet change redox homeostasis and inflammatory conditions, which affect the pathological processes in MetS and related symptoms, thus altering individual miRNA profiles. The relevance of dietary miRNAs in the diet is highlighted by research on their functions, which also provides fresh perspectives on the possible advantages of the foods we eat on a regular basis. These miRNAs can enter the bloodstream and circulate throughout the body, potentially influencing various physiological processes and contributing to the regulation of MetS and its components by modulating gene expression and cellular functions.

Research suggests that dietary patterns such as breast milk, polyphenols such as curcumin, resveratrol and EGCG, and food and dietary components such as rice, orange juice, and the Mediterranean diet may influence miRNA expression and contribute to the prevention of MetS, indicating their potential use as therapeutic targets in supporting metabolic health.

In this context, understanding the complexities of diet–gene interactions and determining lifestyle and dietary habits that affect miRNA expression will enable us to exploit the potential of precision nutrition more effectively in preventing and treating chronic diseases such as MetS. This approach, which combines an understanding of diet and gene interactions with advances in molecular nutrition, may allow for personalized solutions that consider individual differences rather than generic interventions.

Although the clinical use of these molecules as biomarkers and their relationship with nutrition-based interventions are promising, further research is needed on their bioavailability, metabolism, and mechanisms of action. In the future, developing new technologies to increase the bioavailability of dietary miRNAs may enable the use of these molecules as personalized nutrition strategies in the prevention and treatment of metabolic diseases. Future research should focus on increasing our understanding of how different nutrients modulate miRNA profiles, and how individual genetic and environmental factors contribute to these effects. In addition, the development of miRNA-based treatment approaches and the investigation of the therapeutic potential of these molecules in clinical nutrition are important research areas that need to be focused on in the future. In particular, their effects on lipid metabolism, inflammation, and insulin sensitivity may open a new window for disease-specific intervention strategies. Also, personalized nutrition applications based on the analysis of miRNA expression profiles according to the genetic background, metabolic status, and environmental factors of individuals (precision nutrition) may be an important area of research in the future. With this approach, it will be possible to develop more effective nutrition strategies to prevent and manage metabolic diseases such as obesity, type 2 diabetes, cardiovascular diseases, and MetS.

## Figures and Tables

**Table 1:** Key miRNAs affecting components of MetS.

miRNAs	Target	MetS Component	Mechanism of Action	Ref.
miR-132	CREB	Glucose homeostasis	Regulates neural signaling for glucose homeostasis	[9]
miR-9	One Cut Homeobox 2	Insulin secretion	Modulates pancreatic beta-cell function	[10]
miR-124a	Forkhead Box A2	Insulin secretion	Affects beta-cell insulin secretion	[10]
miR-375	Insulin exocytosis proteins	Insulin secretion	Regulates insulin exocytosis	[11]
miR-146a	NF-κB, insulin secretion modulators	Insulin secretion, Inflammation	Regulates immune response and β-cell function	[99]
miR-let-7b	Myotrophin	Glucose regulation	Represses myotrophin	[11]
miR-30d	Insulin gene	Insulin gene expression	Upregulated by glucose, promotes insulin gene	[12]
miR-29	IRS-1, PI3K	Insulin resistance	Affects insulin signaling pathway	[106]
miR-320	IGF-1R	Insulin resistance	Modulates insulin signaling	[106]
miR-150	β-cell function	T2DM	Implicated in T2DM pathogenesis	[5]
miR-143	Adipocyte differentiation genes	Adipogenesis	Promotes adipocyte differentiation	[13]
miR-103	Pparγ2, Fabp4, Glut4	Adipogenesis	Enhances key adipogenic markers	[15]
miR-17-92	Retinoblastoma 2/p130	Adipogenesis	Inhibits p130 to promote differentiation	[16]
miR-122	RAS, endothelial, fibrogenesis pathways	Hypertension, Dyslipidemia	Modulates RAS and fibrogenesis pathways	[18,31]
miR-181	Blood pressure regulators	Blood pressure	Targets genes involved in blood pressure regulation	[21]
miR-663	Renin	Blood pressure	Reduces renin gene expression	[21]
miR-155	TGF-β pathway	Hypertension	Modulates TGF-β signaling affecting cardiac hypertrophy and hypertension	[22]
miR-27a/b	Lipid metabolism genes, PPARγ	Lipid metabolism	Modulates adipogenesis and lipid homeostasis	[100]
miR-34a	SIRT1, lipid metabolism genes	Cardiovascular disease, fatty liver disease	Promotes endothelial senescence; regulates lipid metabolism	[100,108]
miR-126	Endothelial function genes	Atherosclerosis	Reduced levels associated with endothelial dysfunction	[113]
miR-1	Heart-specific expression	Cardiovascular disease	Elevated after myocardial infarction	[113]
miR-133	Cardiac muscle function	Cardiovascular disease	Cardiac-specific, elevated in myocardial injury	[113]
miR-208	Cardiac muscle expression	Cardiovascular disease	Biomarker of cardiac injury	[114]
miR-499	Cardiac muscle expression	Cardiovascular disease	Biomarker of cardiac injury	[114]

**Table 2:** The changes in miRNAs according to nutritional interventions, diet and lifestyle factors.

Intervention	Model	Upregulated or Downregulated miRNAs	Results
18-month hypocaloric diet and physical activity intervention Diet 1: a low-fat diet Diet 2: Mediterranean/low-carbohydrate diet [178]	Adults with abdominal obesity	miR-99-5p/100-5p (↓)	Reduced visceral adiposity and intrahepatic fat accumulation
16-week diet Diet 1: moderately high-protein (30% of total energy from protein) diet Diet 2: low-fat (22% of total energy from fat) diet [189]	Obese subjects (BMI: 30–40 kg/m^2^)	miR-15a-5p (↓) miR-130a-3p (↓) miR-144-5p (↓) miR-221-3p (↓) miR-142-5p (↓) miR-22-3p (↓) miR-29c-3p (↓)	Regulation of lipolysis in fat cells
12-month dietary intervention or exercise program [190]	Overweight and obese postmenopausal women	miR-122 (↓)	Body weight loss
12-week energy-restricted diet (fiber-containing dietary supplements or matched placebo) [182]	Subjects with obesity (BMI: 28–45 kg/m^2^)	miR-122-5p (↓) miR-126a-3p (↑) miR-193a-5p (↓) miR-222-3p (↑)	Adipogenic differentiation Wnt signaling pathway activation is suppressed
8-week Mediterranean diet and physical activity [191]	Girls (age 7–16 years) with abdominal obesity	miR-221-3p (↓)	Reduction in anthropometric indicators such as waist circumference, body weight, and BMI
4-week hypocaloric diet [192]	Obese women (BMI: >30 kg/m^2^)	miR-375 (↑) miR-499 (↑) miR-376 (↑) miR-126 (↑) miR-34a (↓) miR-181a (↓) miR-193a (↓) miR-208 (↓) miR-320 (↓) miR-433 (↓) miR-568 (↓)	Changes in body weight, BMI, blood pressure and serum glucose
2-year energy-restricted diet Diet 1: 20% fat, 15% protein, and 65% carbohydrate Diet 2: 20% fat, 25% protein, and 55% carbohydrate Diet 3: 40% fat, 15% protein, and 45% carbohydrate Diet 4: 40% fat, 25% protein, and 35% carbohydrate [193]	Overweight and obese adults (BMI: 25–40 kg/m^2^)	miR-128-1-5p (↑)	improved adiposity, insulin sensitivity, and energy metabolism
2-month Brazil nut (approximately 1261 μg/Se) [188]	Obese women with and without MetS (BMI: ≥27.5 kg/m^2^)	miR-375 (↓) miR-454-3p (↑) miR-584-5p (↑)	association between calcium homeostasis, vitamin D metabolism, and high Se intake
Plant-based protein (soy protein isolate) or animal-derived protein (calcium caseinate) in a single dose (1 g/kg protein) [194]	Subjects with obesity and IR (BMI: ≥30 kg/m^2^)	miR-27a-3p (↑) miR-29b-3p (↑) miR-122-5p (↑)	Elevated levels of insulin, branched-chain amino acids, and amino acids involved in gluconeogenesis (with consumption of plant-based protein)
Green tea capsule and high-fat, high-saturated fat meal [187]	Obese women (BMI: ≥30 kg/m^2^)	miR-192-5p (↑) miR-373-3p (↑) miR-595 (↑) miR-1266-5p (↑) miR-1297 (↑)	Inhibition of genes associated with adipogenesis and carcinogenesis

(↓) shows that the expression of the relevant miRNA decreases, (↑) shows that the expression of the relevant miRNA increases.

**Table 3:** Association between miRNAs and lipid-related biomarkers in diverse populations with MetS components.

Population	Circulating System	miRNA	Lipid-Related Biomarker	Reference
Dyslipidemia and Hyperglycemia/Young, adult and elderly	Serum	miR-146a, miR-21	TC (+) TG (+) LDL-C (+)	[237]
Newly diagnosed T2DM/Adults and elderly	Plasma	miR-28	TC (+) LDL-C (−)	[257]
Obesity/Young	Serum	miR-122 *	TG (+) HDL-C (−)	[258]
MetS/Children	Plasma	miR-126	TG (+) VLDL-C (+)	[259]
Obesity/Children	Plasma	miR-486 *, miR-130b *, miR-22 *	HDL-C (−)	[260]
Overweight and obesity/Adolescents	Plasma	miR-140, miR-532, miR-22 *, miR-143, miR-423, miR-146a, miR-130b *, miR-142	TG (+) LDL-C (+) HDL-C (+)	[174]

* MicroRNA sequence -3p or -5p.
